# Determinants of non-completion of sleep apnea testing during pregnancy

**DOI:** 10.3389/frsle.2023.1144213

**Published:** 2023-03-27

**Authors:** Kathleen M. Antony, Alexandra Lauren Rice, Sakshi Bajaj, Abigail M. Wiedmer, Natalie Jacobson, Julia Nick, Allison Eichmann, Aleksandar K. Stanic, Mihaela H. Bazalakova

**Affiliations:** ^1^Division of Maternal-Fetal Medicine, Department of Obstetrics and Gynecology, School of Medicine and Public Health, University of Wisconsin-Madison, Madison, WI, United States; ^2^Department of Biology, University of Wisconsin-Madison, Madison, WI, United States; ^3^Department of Neurology, Wisconsin Sleep, Wisconsin Institute for Sleep and Consciousness, School of Medicine and Public Health, University of Wisconsin-Madison, Madison, WI, United States; ^4^Division of Reproductive Sciences, Department of Obstetrics and Gynecology, School of Medicine and Public Health, University of Wisconsin-Madison, Madison, WI, United States

**Keywords:** pregnancy, health care access, sleep test completion, no-show, public insurance, sleep clinic, obstetrics, sleep apnea

## Abstract

**Study objectives:**

Completion of testing during pregnancy for those who screen positive for obstructive sleep apnea (OSA) is imperative for the diagnosis and treatment of OSA, as the latter may reduce the risk of developing hypertensive disorders of pregnancy. To identify potential barriers, we assessed predictors of non-completion of sleep apnea testing by people identified to be at high risk of OSA by screening during pregnancy. We hypothesized that non-completion of sleep apnea testing would be predicted by insurance status and obstetric factors, such as gestational age at time of testing.

**Methods:**

We performed a retrospective analysis of the first 500 people in our sleep pregnancy database which includes both pregnant and preconception patients who screened positive for OSA; those screened preconception were excluded. Multivariable Poisson regression was used to determine which factors were independently associated with non-completion.

**Results:**

Of 445 referred, 214 (48.1%) completed sleep apnea testing. Factors associated with non-completion of testing on univariate analysis included referral in the third trimester, higher parity, one or more living children, history of preterm birth, history of preeclampsia, type 2 diabetes mellitus, non-partnered status, race, and payor. Symptoms of loud snoring or witnessed apneas were associated with increased incidence of sleep apnea testing completion. Multivariable Poisson regression demonstrated that having public insurance predicted non-completion of sleep apnea testing during pregnancy.

**Conclusion:**

In this small study, public insurance was an independent predictor of non-completion of sleep apnea testing during pregnancy. These findings aid efforts to improve patient completion of sleep apnea testing during pregnancy.

## Introduction

Obstructive sleep apnea (OSA) is a risk factor for gestational hypertension and preeclampsia (hypertensive disorders of pregnancy), even in pregnancies not complicated by obesity or medical comorbidities (Facco et al., 2017). While estimates of OSA incidence in pregnant women are likely to vary depending on gestational age at time of testing (Sanapo et al., 2021), diagnostic modalities (Antony et al., 2014; Facco et al., 2014), criteria (Khalid et al., 2021) and comorbidities, OSA has been reported in up to 9% of early, low risk, non-obese (Facco et al., 2017) and up to 60% of high risk pregnancies complicated by obesity and/or chronic hypertension (Facco et al., 2014; O'Brien et al., 2014; Antony et al., 2021; Johns et al., 2022). There is emerging evidence that treatment of OSA with continuous positive airway pressure (CPAP) may reduce the incidence of hypertensive disorders of pregnancy, including preeclampsia (Stajić et al., 2021; Rice et al., 2022). OSA thus presents a novel, potentially modifiable, prevalent risk factor for preeclampsia—a leading cause of maternal morbidity and mortality, with limited options for prevention and treatment (Edwards et al., 2000; Blyton et al., 2004; Poyares et al., 2007; Pamidi et al., 2014; Bazalakova, 2017). However, either in-lab polysomnography (PSG) or a home sleep apnea test (HSAT) is required in the clinical setting for the diagnosis and treatment of OSA.

The no-show rate to sleep clinic appointments in the general population is estimated to be as high as 20% (Cheung et al., 2020). Completion of sleep clinic appointments and sleep apnea testing while pregnancy is ongoing may be even lower due to appointment delays and, potentially, pregnancy- or parenting-specific factors, such as the need to arrange for childcare in order to be able to complete overnight in-lab PSG testing, or to pick up and return HSAT equipment from a specialty sleep clinic.

We sought to analyze potential predictors of sleep apnea testing completion during pregnancy. Our hypothesis was that non-completion of testing could potentially be predicated by obstetric factors and insurance status. We plan to use information about these predictors of non-completion to target interventions in order to improve access to sleep apnea testing during pregnancy.

## Methods

### Clinical design and data

This retrospective cohort study draws from a collaborative clinical and research pipeline between the Division of Maternal-Fetal Medicine at the University of Wisconsin-Madison/UnityPoint Health-Meriter and the Wisconsin Sleep Clinic at the University of Wisconsin-Madison. MHB and KMA created a cross-disciplinary Sleep Pregnancy Clinic, which facilitates streamlined screening, diagnosis, and treatment of OSA during pregnancy (Antony et al., 2021). This retrospective cohort study was reviewed and approved by the UnityPoint Health-Meriter's Institutional Review Board (2019-014).

Pregnant people who present with pre-pregnancy body mass index (BMI) ≥30 kg/m^2^ or who self-report snoring or other sleep complaints, are screened for OSA at their initial prenatal care visit using a simple, four variable pregnancy-specific questionnaire developed by Facco et al. (2012) (Antony et al., 2021). In this tool, age and body mass index (BMI) are added, and 15 points each are added also if the woman has chronic hypertension or snores >3 nights per week. A screening score greater than or equal to 75 results in referral to sleep clinic for sleep apnea evaluation.

We chose Facco et al.'s (2012) four variable tool because, of the available tools to screen for sleep apnea, it has the highest area under the receiver operating characteristic curve in pregnancy, including among pregnant people with BMI ≥40 kg/m^2^ (Dominguez et al., 2018). Additionally, maternal age, BMI, and frequent snoring—three of the variables used by this screening tool—have been identified as the most reliable predictors of OSA diagnosis in pregnancy in the breathing substudy of the nuMoM2b, the largest prospective study of OSA prevalence and incidence in nulliparous normal pregnancies (Louis et al., 2018). Facco et al.'s (2012) four variable tool is also reasonably simple to use, only requiring one subjective symptom report, that of frequent snoring (Antony et al., 2021). In our clinic, as noted above, all pregnant people with a pre-pregnancy BMI ≥30 kg/m^2^ or who self-report snoring or other sleep complaints, are screened for OSA.

Once a pregnancy referral is received by the sleep clinic, it is further triaged either directly for home sleep apnea testing with the Respironics Alice PDx^®^, which is currently in routine clinical use at the Wisconsin Sleep Clinic for both pregnant and non-pregnant patients^6^, or to sleep clinic visit first, in cases where a face-to-face visit for documentation of symptoms or exam is required by insurance. The default is to triage pregnancy referrals to sleep apnea testing directly, in order to expedite the diagnostic and therapeutic processes given the time limitations of pregnancy, as wait times for sleep clinic appointments are typically several weeks to months. Exceptions are made in the minority of cases where patients need to be evaluated in sleep clinic first, because of missing referral documentation of symptoms or exam, or complicated clinical presentations (Antony et al., 2021). In this analysis, 9% (*n* = 42) vs. 91% (*n* = 403) out of the 445 total referrals were triaged to a sleep clinic visit vs. sleep apnea testing, respectively.

The STOP-BANG questionnaire (Chung et al., 2008, 2012; Antony et al., 2021) is also queried at the prenatal visit, and responses are sent to the Wisconsin Sleep Clinic, but referral decisions are made based upon responses to the pregnancy-specific four variable tool (Facco et al., 2012). Those who are referred for sleep testing are provided with a handout indicating common symptoms of OSA, complications of pregnancy that are associated with OSA, and pictures of pregnant people wearing both the sleep testing equipment and a CPAP machine. Patients are referred by multiple clinicians spanning six academic obstetrics and gynecology clinics, and four distinct community obstetrics and gynecology groups, in addition to midwifery groups and family and community medicine groups across the Madison area.

For the purposes of this study, those whose race was categorized as “American Indian and Alaska Native,” “Native Hawaiian,” and “Other” were combined due to low numbers to protect anonymity. “Partnered” marital status included those who described their marital status as “married,” “partnered,” or having a “significant other.” “Non-partnered” marital status was defined as “single,” “divorced,” “separated,” or “widowed.”

Chronic hypertension and pre-pregnancy diabetes were defined in accordance with the guidelines of the American College of Obstetricians and Gynecologists (ACOG) (The American College of Obstetricians Gynecologists, 2018; ACOG Committee on Practice Bulletins—Obstetrics, 2019). Gestational weight gain was calculated utilizing the woman's weight at delivery and subtracting pre-pregnancy or early pregnancy weight, as is commonly used in both clinical and research settings (Johnson et al., 2013; Kominiarek and Peaceman, 2017; Voerman et al., 2019; Antony et al., 2020). Gestational weight gain was categorized as insufficient, appropriate, or excess using formulas derived from the Institute of Medicine corrected for the gestational age at delivery (Committee to Reexamine IOM Pregnancy Weight Guidelines, 2009; Johnson et al., 2013). A composite of medical comorbidities variable was also created to estimate the burden of chronic illnesses and likely multiple medical appointments. This variable assigned 1 point for each of the following medical conditions and was summed to tabulate the total number of comorbidities: chronic hypertension, type 1 diabetes, type 2 diabetes, hyperthyroidism, hypothyroidism, asthma, depression, anxiety, heart failure, polycystic ovarian syndrome, smoking, alcohol, illicit substance use, arrhythmias, and history of venous thromboembolism. While this is not a validated measure, this variable was created to evaluate whether a high burden of likely other medical appointments impacted sleep apnea test completion, either positively or negatively.

### Data collection and extraction

A Health Insurance Portability and Accountability Act (HIPAA)-compliant, institutional review board (IRB)-approved (2019-014) database of all people who screened positive for OSA during pregnancy or at a preconception visit and were referred for HSAT or sleep clinic evaluation was created for quality improvement clinical and research purposes (Antony et al., 2021). This database was maintained in Research Electronic Data Capture (REDCap) (Harris et al., 2009, 2019) a secure online database used by the UW Institute for Clinical and Translational Research (UW ICTR) for data collection in clinical research. IRB approval was obtained for this retrospective cohort study using this dataset. In this database, demographic, insurance, obstetric, medical, and pregnancy outcome information was manually extracted from the electronic health record of UW Health (Healthlink Epic) and UnityPoint Health (Epic, Hyperspace 2017). Data regarding whether sleep apnea testing was completed and the test results, treatment recommendations, and treatment adherence and compliance metrics were also recorded. Data on the first 500 people entered into the database were queried for this analysis.

Exclusion criteria for this present analysis were those screened for OSA at a preconception consultation and referred to the Sleep Pregnancy Clinic or triaged to HSAT prior to being pregnant. Inclusion criteria were those who screened positive for being high risk for potentially having OSA during pregnancy.

Those who completed sleep apnea testing were compared to those who did not. Demographic and obstetric characteristics and outcomes were assessed using Pearson's chi-square, Fisher's exact test, Student's *T*-test, and Wilcoxon-Rank sum where appropriate. Parity is not continuous because all values are integers, but for the purposes of data display, both parametric and non-parametric data are displayed for clarity. P < 0.05 was considered to be statistically significant. Relative risk and adjusted relative risk were obtained using Poisson regression. All statistical analyses were performed using STATA 16.0 (StataCorp, 2017, College Station, TX).

## Results

The first 500 people were referred to the sleep pregnancy clinic between June 1, 2017 and February 4, 2022. 55 of these people were referred following a preconception consultation and were excluded due to not being pregnant at the time of referral. Accordingly, 445 pregnant people were referred for OSA evaluation and testing. Of these 445 referrals, 214 (48.1%) completed sleep testing during pregnancy.

As shown in Table 1, Black or African American race and “Other, unknown, or not reported” race, public insurance, and non-partnered status were associated with sleep apnea testing non-completion. Medical co-morbidities, obstetric characteristics, and specific sleep symptoms were also associated with non-completion of a sleep apnea testing during pregnancy. A history of type 2 diabetes, higher parity or at least one living child, history of prior preterm birth, and history of preeclampsia in a prior pregnancy were associated with sleep apnea testing non-completion. Referral later in pregnancy was also associated with testing non-completion. Loud snoring and witnessed apneas were associated with completion of sleep apnea testing, but daytime tiredness, as queried in the STOP-BANG, and frequent snoring were not.

**Table 1:** Demographic, medical, and obstetric characteristics.

**Characteristic**	**Sleep test not completed**	**Sleep test completed**	**Relative risk**	***p***
	***N*** = **229**	***N*** = **214**	**(95% CI)**	
Age at referral, mean (SD)	32.1 (5.5)	32.9 (5.6)	–	0.123
Age >35 at referral, *n* (%)	89 (38.5)	92 (43.0)	1.10 (0.91–1.34)	0.338
**Race/Ethnicity**, ***n*** **(%)**				0.007
White/Caucasian	142 (61.5)	161 (75.2)	Ref	
Black/African American	45 (19.5)	21 (9.8)	0.60 (0.41–0.87)	
Hispanic/Latinx	14 (6.1)	16 (7.5)	1.00 (0.71–1.43)	
Asian or Indian	8 (3.5)	6 (2.8)	0.81 (0.44–1.49)	
Other, unknown, or not reported^*^	22 (9.5)	10 (4.7)	0.59 (0.35–0.99)	
**Payor**, ***n*** **(%)**^†‡^				0.010
Private	129 (55.8)	147 (69.7)	Ref	
VA/Tricare	3 (1.3)	1 (0.5)	0.47 (0.09–2.58)	
Public	93 (40.3)	56 (26.5)	0.71 (0.56–0.89)	
No active insurance	6 (2.6)	7 (3.3)	1.01 (0.60–1.69)	
Partnered^¶**^	113 (51.8)	153 (73.2)	1.64 (1.25–2.15)	<0.001
**Medical co–morbidities**				
Prepregnancy BMI, median (IQR)	40.7 (34.6–46.5)	40.0 (34.8–46.6)	–	0.972
Chronic hypertension, *n* (%)^††^	79 (34.5)	66 (31.3)	0.93 (0.75–1.15)	0.473
Type 2 diabetes, *n* (%)^‡‡^	46 (20.3)	28 (13.2)	0.75 (0.55–1.02)	0.048
Hypothyroidism, *n* (%)	22 (9.8)	31 (14.8)	1.24 (0.97–1.60)	0.116
Total number of comorbidities, median (IQR)^§§^	2 (1–3)	2 (1–3)	–	0.815
**Prior obstetric history**				
Parity, median (IQR)	1 (0–2)	1 (0–2)	–	<0.001
Parity, mean (SD)^¶¶^	1.7 (1.5–1.9)	1.1 (0.9–1.3)	–	<0.001
One or more living children, *n* (%)	169 (73.2)	137 (64.0)	0.81 (0.66–0.98)	0.038
Prior gestational diabetes^***^	6 (13.6)	18 (9.3)	0.81 (0.72–1.16)	0.380
Prior preterm birth	45 (19.5)	23 (10.8)	0.67 (0.47–0.94)	0.011
Prior preeclampsia^***^	15 (34.1)	31 (15.9)	0.79 (0.64–0.98)	0.006
**Obstetric characteristics**				
Gestational age at referral, median (IQR)	15.4 (11.6–24.9)	14 (9.3–21.0)	–	0.003
Trimester at referral				0.023
First trimester	99 (42.9)	106 (49.5)	Ref	
Second trimester	87 (37.7)	86 (40.2)	0.96 (0.79–1.17)	
Third trimester	45 (19.5)	22 (10.3)	0.64 (0.44–0.92)	
**Sleep symptoms**				
Frequent snoring, *n* (%)	151 (66.2)	158 (74.5)	1.01 (1.00–1.03)	0.057
Loud snoring, *n* (%)	109 (57.1)	139 (70.6)	1.35 (1.08–1.70)	0.006
Tiredness or fatigue *n* (%)^†††^	133 (69.6)	130 (66.0)	0.92 (0.75–1.13)	0.443
Witnessed apneas, *n* (%)	27 (14.5)	44 (22.7)	1.28 (1.03–1.58)	0.041

^*^Other race includes American Indian and Alaska Native, Native Hawaiian, and Other. Data combined due to low numbers to protect anonymity.

^†^*P*-value from Fisher's exact test.

^‡^Insurance data not available for 3 people.

^¶^Partnered defined as married, partnered, or significant other.

^**^Data not available for 18 people.

^††^Data not available for 5 people.

^‡‡^Data not available for 6 people.

^§§^This variable assigned 1 point for each of the following medical conditions and was summed to tabulate the total number of comorbidities: chronic hypertensin, type 1 diabetes, type 2 diabetes, hyperthyroidism, hypothyroidism, asthma, depression, anxiety, heart failure, polycystic ovarian syndrome, smoking, alcohol, illicit substance use, arrhythmias, and history of venous thromboembolism.

^¶¶^Not continuous but shown for clarity purposes.

^***^Only reported for those with prior deliveries.

^†††^Tiredness item is derived from the STOP BANG questionnaire.

We also assessed whether completion of a sleep clinic visit was associated with completion of sleep apnea testing. Of the 42 gravidae who completed a sleep clinic visit prior to a sleep test being scheduled, 32 (76.2%) completed sleep apnea testing. In comparison, 182 from 403 gravidae (45.2%) who were triaged directly to sleep apnea testing and did not complete a sleep clinic visit first, completed sleep apnea testing (*P* < 0.001).

In order to assess whether the COVID pandemic affected HSAT completion rates, we compared referrals and completed HSATs for the 9 months prior to (April 1–December 31, 2019) vs. following (April 1-December 31, 2020) COVID-related changes in clinical care; of note, Wisconsin Sleep suspended in-person clinic visits and PSG sleep studies on March 13, 2020. We noted a 26% drop in referrals during the 9 month period post-COVID compared to pre-pandemic (85 vs. 115 referrals), however the rates of HSAT completion remained similar, at 56.5 and 52%, respectively. We suspect the slightly higher rate of HSAT completion during this time period compared to the overall average may be related to the growing visibility, familiarity with, and utilization of the sleep pregnancy clinical pipeline by both clinicians and patients.

Multivariate Poisson regression analysis, incorporating the significant univariate variables of race, payor, partnered status, type 2 diabetes, one or more living children, prior preterm birth, prior preeclampsia, loud snoring, and witnessed apneas, demonstrated that only having a public payor was independently predictive of sleep apnea testing non-completion during pregnancy in our cohort (Table 2). Hispanic/Latinx ethnicity was independently predictive of sleep apnea testing completion.

**Table 2:** Multivariate poisson regression predicting non-completion of sleep test incorporating the significant univariate independent variables.

**Characteristic**	**Adjusted relative risk**
	**(95% CI)**
**Race/Ethnicity**, ***n*** **(%)**	
White	Ref
Black	1.07 (0.84–1.36)
Hispanic/Latinx	1.20 (1.03–1.40)
Asian	0.81 (0.49–1.35)
Other, unknown, or not reported	0.92 (0.68–1.25)
**Payor**, ***n*** **(%)**	
Private	Ref
VA/Tricare	0.51 (0.13–2.03)
Public	0.79 (0.67–0.93)
No active insurance	1.06 (0.88–1.28)
Partnered	1.07 (0.88–1.30)
**Medical co–morbidities**	
Type 2 diabetes, *n* (%)	0.81 (0.63–1.03)
**Prior obstetric history**	
One or more living children, *n* (%)	0.97 (0.87–1.08)
Prior preterm birth	0.96 (0.75–1.22)
Prior preeclampsia	0.85 (0.69–1.05)
**Obstetric characteristics**	
Trimester at referral	
First trimester	Ref
Second trimester	1.00 (0.81–1.24)
Third trimester	0.85 (0.54–1.35)
**Sleep symptoms**	
Loud snoring, *n* (%)	0.95 (0.83–1.08)
Witnessed apneas, *n* (%)	1.10 (0.94–1.28)

We also calculated the average time from referral to sleep apnea test completion for those who completed a sleep study. The average delay was 43.9 days with a 95% confidence interval from 35.3 to 52.5 days.

## Discussion

In this analysis, we found that 51.9% of pregnant people who were referred for OSA evaluation and testing during pregnancy did not complete the recommended sleep apnea testing. Our setting is a university-based specialty clinic with a sleep-pregnancy clinic, which is a specific referral pipeline with a sleep physician “champion” who assists with prioritizing OSA evaluation and testing for pregnant people to minimize delays, thus this referral completion rate may be higher than would be expected in other settings. Indeed, the time from referral to HSAT completion was an average of 43.9 days, compared to 2–10 months in the general non-pregnant population (Flemons et al., 2004; Sriram et al., 2021). However, we also have barriers that may be specific to our setting, such as the sleep clinic being at a different location than the obstetric clinics, and our workflow where patients pick up and return portable HSAT equipment from the sleep clinic, without the option of equipment being mailed to the patient and then mailed back to the sleep center.

Regarding predictors of sleep apnea testing non-completion, we found that having public insurance was independently associated with higher rates of non-completion. Our population had a low prevalence of pregnant people with no insurance. Studies in the non-pregnant population have found private insurance to be associated with higher sleep appointment completion and lack of insurance to be associated with “no-shows” to sleep clinic appointments (Cheung et al., 2020). Public or non-commercial insurance has also been associated with higher “no-show” rates at academic pain clinics and gastrointestinal clinics (Odonkor et al., 2017; Shuja et al., 2019). Public insurance is associated with other barriers to healthcare access that were not fully queried in this evaluation such as access to transportation, childcare to cover clinical appointments, and available time off from work. Access to reliable transportation is now routinely queried upon entry to prenatal care, but in this dataset, including data from 2017 onward, this variable was not routinely queried and thus could not be incorporated into our analysis. Access to childcare or time off from work are not routinely queried as part of prenatal care, but they could also impact sleep apnea testing completion or sleep clinic visit if required pre-testing. Notably, pregnant people whose public insurance is provided only because they are pregnant often lose this coverage postpartum, so if a sleep apnea test is not completed during pregnancy, the opportunity to complete this diagnostic testing and obtain treatment may be missed. This is also unfortunate as OSA likely poses ongoing health risks after pregnancy is completed (Javaheri et al., 2017).

Hispanic or Latinx ethnicity was independently associated with increased completion of sleep testing during pregnancy. The reasons for this are unclear, but whatever protective factors are effective in this population would be worth investigating in other populations. Qualitative research may help elucidate these protective factors.

On univariable analysis, several other factors were also associated with non-completion of sleep apnea testing. Possible reasons for higher rates of non-completion for higher gestational age may be that the pregnancy ended prior to completion of the sleep test due to delivery, a pregnancy complication arising, or patient-perceived futility in pursuing evaluation late in pregnancy. The average referral date to sleep test completion date was ~6 weeks, and many in the third trimester may have delivered in such a timeframe. Advanced gestational age can also be associated with poorer sleep quality and more physical discomfort, and those referred in the third trimester may not feel as comfortable undergoing an overnight sleep apnea test or may lack buy-in or familiarity with OSA as a potential etiology of sleep disturbance. As for parity, higher parity would be expected to correlate with having more living children, often young children, living at home which may preclude attendance to overnight in-lab PSG or sleeping arrangements at home conducive to HSAT completion.

We also looked at specific sleep symptoms, which may impact the pregnant person's sense of urgency about completing sleep apnea testing. While frequent snoring was not significantly associated with completion of testing, loud snoring and witnessed apneas were. This may suggest that those with sleep partners who are aware of and can report sleep symptoms may encourage testing; indeed, a self-described relationship status as “partnered” was associated with increased completion of sleep testing. Witnessed apneas may also lead to a sense of concern and fear and prompt a sleep test. Unfortunately, patients and clinicians may be falsely reassured by absence of witnessed apneas which, despite “apnea” misleadingly featuring in the name of the disorder, are not in fact a requisite symptom of OSA.

We also found that a history of preeclampsia in a prior pregnancy was associated with a lower, rather than higher, completion of sleep testing. Thus, those who were at the highest risk of developing preeclampsia and potentially benefitting from diagnosis and treatment of OSA (if diagnosed) were not obtaining the diagnostic test which could potentially reduce this risk. This finding may speak to the importance of clinician education on the possible medical benefits of OSA diagnosis and treatment during pregnancy, which in turn could inform patient-clinician discussions and joint decision-making prioritization.

Our study has several strengths. While prior studies have evaluated no-show rates to academic medical clinics (Odonkor et al., 2017; Shuja et al., 2019), and one has reported on predictors of no-show rates to sleep clinics (Cheung et al., 2020), none have focused on pregnant patients or on factors which may be specific to a pregnant population. Because completion of sleep apnea testing is necessary to diagnose and initiate treatment of OSA, untreated OSA is a risk factor for preeclampsia (Ursavas et al., 2008; Louis et al., 2010; O'Brien et al., 2014; Pamidi et al., 2014; Bazalakova, 2017; Facco et al., 2017; Dominguez et al., 2018), and CPAP treatment may reduce the risk of developing preeclampsia, failure to complete testing and diagnose OSA in pregnancy may impact the likelihood of developing preeclampsia (Stajić et al., 2021; Rice et al., 2022). We have identified potential predictors of non-completion of sleep apnea testing, namely public insurance, which could be used to target interventions to improve testing completion. Another strength of our study, is that we used a database that we created specifically to track patient referrals and follow-up. Data were extracted manually from the electronic health record by trained researchers with audit and oversight by board-certified sleep and maternal-fetal medicine physicians (MHB and KMA, respectively).

This study also has limitations. While pregnancy-unique factors of clinical importance, such as advanced gestational age, were identified as risk factors for sleep apnea testing non-completion, those were not significant in multivariable regression analysis. The relatively small sample size may have underpowered our multivariable analysis.

Inherent to all retrospective assessments based upon abstraction of electronic health records, information that was not collected at the time of clinical care could not be abstracted. Notably, our pregnant patients now undergo surveys on social determinants of health at the time of clinic intake. This information includes whether the patient experiences barriers to transportation access which would likely impact ability to travel to the sleep clinic for and insurance-mandated clinic visit, or the sleep lab for overnight PSG, or to the sleep clinic and back to retrieve and return HSAT equipment. This, and other unmeasured factors that can be associated with insurance status, such as income and, as noted above, access to reliable transportation, likely contributed to unmeasured confounding effects that could not be fully accounted for.

We also specifically evaluated sleep apnea testing completion rather than sleep clinic or sleep testing appointment no-show. Our interest here was in completion of the sleep apnea testing because this is the data point needed for diagnosis and treatment. We did not separately examine individuals who were referred to the sleep clinic or for sleep testing, but did not complete the appointment or those who never made appointments. While the potential psychosocial rationale between not showing for an appointment and not making an appointment may differ, we did not delve into predictors of these non-completion points because our interest was in completion of sleep apnea testing, as above.

Our triage system allowed patients to directly proceed to home sleep testing, thus not all patients needed to be seen in clinic prior to their home sleep test. However, among those seen in clinic prior to a sleep test being scheduled, 76.2% completed their sleep test compared to only 45.2% of those triaged straight to home sleep testing. While a sleep clinic visit requirement prior to sleep apnea testing may be a barrier to completion of testing due to long wait times for clinic appointments, it may also be that the discussion and educational value of a sleep clinic visit encourages patients and increases likelihood of compliance with sleep apnea testing completion. We found that sleep clinic visit prior to the sleep test was associated with a higher sleep test completion rate. However, patients were already at the visit and could easily retrieve the test. Requiring such a visit, which can have lengthy delays at a busy academic clinic, may or may ultimately improve sleep test completion rates overall.

One interesting finding was that, while referrals dipped during the beginning of the COVID pandemic, HSAT completion rates remained relatively stable. Of note, our clinical center resumed HSATs earlier than PSGs, especially titration studies. Thus, the sustained HSAT completion rates in our population points to a potential unexpected advantage of home studies in the unique situation of an infectious disease public health emergency.

While the efficacy of CPAP at reducing the incidence of hypertensive disorders of pregnancy remains to be demonstrated in a randomized trial, evidence increasingly supports the role CPAP treatment of OSA in pregnancy may play in the prevention and treatment of preeclampsia (Edwards et al., 2000; Blyton et al., 2004; Poyares et al., 2007; Pamidi et al., 2014; Whitehead et al., 2015; Bazalakova, 2017) a major source of maternal morbidity and mortality without current prevention or treatment interventions. Two small studies, one prospective non-randomized and one retrospective, suggest treatment of OSA with CPAP may help prevent preeclampsia or gestational hypertension (Stajić et al., 2021; Rice et al., 2022).

We found that public insurance is an independent predictor of sleep apnea testing non-completion during pregnancy. Future efforts should focus on qualitative assessments of people with public insurance to assess for additional barriers to access to sleep tests. Efforts should also focus on screening early in pregnancy, and the design and implementation of clinical protocols and equipment distribution workflows to reduce identified barriers, such as offering sleep apnea testing equipment out of the obstetric clinic with a sleep physician available *via* telehealth or embedded in the obstetric clinic. Other novel options may include assistance with childcare to allow patients to attend clinic visits, if required. One intervention of value may be and emphasis on education of referring clinicians, including obstetricians, family doctors or other clinicians routinely taking care of gravidae, as to the specific predictors of HSAT non-completion, including public insurance, which we have identified in this study. Early gestational age at time of referral may be a particularly important goal, given wait times for study and/or clinic appointments, CPAP setup and expected greater prevention benefit of CPAP treatment initiated earlier in pregnancy. Further studies are needed to develop methods to improve sleep apnea testing completion targeting those at risk for not completing testing, and to assess whether these efforts resulted in improvement in OSA testing completion, particularly among those most at risk.

## Data availability statement

The raw data supporting the conclusions of this article will be made available by the authors, without undue reservation.

## Ethics statement

The studies involving human participants were reviewed and approved by UnityPoint Health-Meriter's Institutional Review Board (2019-014). Written informed consent for participation was not required for this study in accordance with the national legislation and the institutional requirements.

## Author contributions

KA, AS, and MB: conceptualization and design, data analysis, and interpretation. KA, AR, SB, AW, NJ, JN, AE, and MB: data acquisition. KA and MB: drafted portions of the manuscript and drafted a critical review. All authors contributed to the article and approved the submitted version.
